# Expression, Functional Characterization, and Preliminary Crystallization of the Cochaperone Prefoldin from the Thermophilic Fungus *Chaetomium thermophilum*

**DOI:** 10.3390/ijms19082452

**Published:** 2018-08-19

**Authors:** Kento Morita, Yohei Y. Yamamoto, Ayaka Hori, Tomohiro Obata, Yuko Uno, Kyosuke Shinohara, Keiichi Noguchi, Kentaro Noi, Teru Ogura, Kentaro Ishii, Koichi Kato, Mahito Kikumoto, Rocio Arranz, Jose M. Valpuesta, Masafumi Yohda

**Affiliations:** 1 Department of Biotechnology and Life Science, Tokyo University of Agriculture and Technology, Naka, Koganei, Tokyo 184-8588, Japan; kento.morita@yohda.net (K.M.); yohei.yamamoto@yohda.net (Y.Y.Y.); ayaka.hori@yohda.net (A.H.); tomohiro.obata@yohda.net (T.O.); yuko.uno@yohda.net (Y.U.); k_shino@cc.tuat.ac.jp (K.S.); 2 Instrumentation Analysis Center, Tokyo University of Agriculture and Technology, Naka, Koganei, Tokyo 184-8588, Japan; knoguchi@cc.tuat.ac.jp; 3 Department of Molecular Cell Biology, Institute of Molecular Embryology and Genetics, Kumamoto University, Kumamoto 860-0811, Japan.; noi@abc.me.es.osaka-u.ac.jp (K.N.); ogura@gpo.kumamoto-u.ac.jp (T.O.); 4 Core Research for Evolutional Science and Technology (CREST), JST, Kawaguchi, Saitama 332-0012, Japan; 5 Exploratory Research Center on Life and Living Systems, National Institutes of Natural Sciences, Myodaiji, Okazaki 444-8787, Japan; ishii@ims.ac.jp (K.I.); kkatonmr@ims.ac.jp (K.K.); 6 Structural Biology Research Center, Graduate School of Science, Nagoya University, Chikusa-ku, Nagoya, Aichi 464-8601, Japan; kikumoto.mahito@a.mbox.nagoya-u.ac.jp; 7 Departamento de Estructura de Macromoléculas, Centro Nacional de Biotecnología (CNB-CSIC), Madrid 28049, Spain; rarranz@cnb.csic.es (R.A.); jmv@cnb.csic.es (J.M.V.)

**Keywords:** chaperone, chaperonin, *Chaetomium thermophilum*, proteostasis, interaction, folding

## Abstract

Prefoldin is a hexameric molecular chaperone found in the cytosol of archaea and eukaryotes. Its hexameric complex is built from two related classes of subunits, and has the appearance of a jellyfish: Its body consists of a double β-barrel assembly with six long tentacle-like coiled coils protruding from it. Using the tentacles, prefoldin captures an unfolded protein substrate and transfers it to a group II chaperonin. Based on structural information from archaeal prefoldins, mechanisms of substrate recognition and prefoldin-chaperonin cooperation have been investigated. In contrast, the structure and mechanisms of eukaryotic prefoldins remain unknown. In this study, we succeeded in obtaining recombinant prefoldin from a thermophilic fungus, *Chaetomium thermophilum* (CtPFD). The recombinant CtPFD could not protect citrate synthase from thermal aggregation. However, CtPFD formed a complex with actin from chicken muscle and tubulin from porcine brain, suggesting substrate specificity. We succeeded in observing the complex formation of CtPFD and the group II chaperonin of *C. thermophilum* (CtCCT) by atomic force microscopy and electron microscopy. These interaction kinetics were analyzed by surface plasmon resonance using Biacore. Finally, we have shown the transfer of actin from CtPFD to CtCCT. The study of the folding pathway formed by CtPFD and CtCCT should provide important information on mechanisms of the eukaryotic prefoldin–chaperonin system.

## 1. Introduction

Protein homeostasis is maintained by molecular chaperones [1]. Most molecular chaperones are heat shock proteins (Hsps) that are expressed under stress conditions to protect proteins from thermal denaturation. Molecular chaperones recognize the hydrophobic surface of unfolded or misfolded polypeptides, and induce correct folding or facilitate degradation. Chaperonins, also known as Hsp60s, are the most important and ubiquitous chaperones. Chaperonins exist as two stacked rings, which are composed of 7–9 subunits of approximately 60 kDa each. Chaperonin captures an unfolded protein in the cavity of each ring and mediates protein folding in an ATP-dependent fashion. Chaperonins are subdivided in two families, group I and group II chaperonins [2,3]. Group I chaperonins exist in eubacteria and eukaryotic organelles, such as mitochondria and chloroplasts. Group II chaperonins are found in archaea and eukaryotic cytosol. 

Prefoldin (PFD) is a cochaperone of group II chaperonin. PFD, originally discovered when screening yeast genes, is involved in microtubule formation and acts in an actin-folding complex [4,5], and is also found in archaea. Archaeal PFDs are hexamers composed of two types of subunits: Two α subunits and four β subunits. Archaeal PFDs are molecular chaperones capable of stabilizing a range of nonnative proteins, and releasing them for subsequent group II chaperonin-assisted folding [6,7,8]. Eukaryotic PFD interacts specifically and transfers substrates to a eukaryotic group II chaperonin (chaperonin containing TCP-1 (CCT); also called TCP-1 ring complex (TRiC)). Eukaryotic PFDs are composed of six different subunits: two α-like subunits (PFD3 and PFD5) and four β-like subunits (PFD1, PFD2, PFD4, and PFD6). 

**Figure 1:**
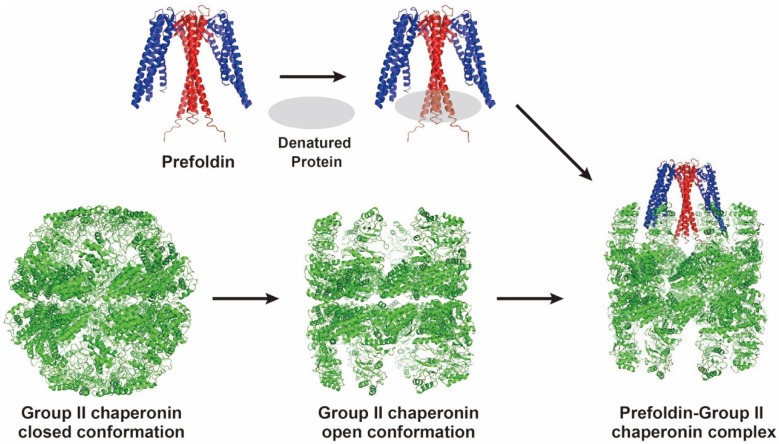
The schematic model for the cooperation between archaeal prefoldin (PFD) and group II chaperonin. Crystal structures of *Pyrococcus* PFD (PDB ID: 2ZDI), and *Methanococcus* group II chaperonins (PDB IDs: 3LOS (closed), 3IYF (open)) are illustrated by PyMOL (Schrödinger Ltd., Cambridge, MA, USA). The α subunit and β subunit of PFD are shown in red and blue, respectively. The complex is also illustrated by PyMOL. PFD captures an unfolded protein by six tentacles, and transfers it to a group II chaperonin in the open conformation.

The crystal structures of archaeal PFDs from *Methanobacterium thermoautotrophicum* and *Pyrococcus horikoshii* OT3 have been determined to 2.3 and 3.0 Å resolution, respectively [9,10]. PFD resembles a jellyfish: Its body consists of a double-barrel assembly with six long tentacle-like coiled coils protruding from it (Figure 1). Each of the α and β subunits contains two or one central β-hairpin, respectively; these are N- and C-terminally flanked by coiled-coil helices. The coiled-coil helices of each subunit assemble into an antiparallel conformation. The detailed interaction mechanism between archaeal PFD and group II chaperonin has been studied (Figure 1) [11,12,13]. 

Archaeal group II chaperonins consist of two rings made of one to three different subunits. In contrast, CCT adopts a complex structure with each ring composed of eight different subunits. PFDs interact and function with group II chaperonins in a cooperative manner. Thus, it is reasonable to think that PFDs coevolved with group II chaperonins. The importance of PFD in proteostasis in the cytosol has been widely recognized [14]. However, there has been little advancement in the study of eukaryotic PFDs due to the complexity of eukaryotic PFDs and their partner CCTs. Simons et al. have shown that a functional eukaryotic PFD can spontaneously assemble from its six constituent individual subunits [15]. Using engineered forms of PFD assembled in vitro, they showed that the tips of PFD tentacles are required to form binary complexes with authentic target proteins. They also presented data that suggest a model for the assembly order of these six subunits within the hexamer. Although cryo-electron microscopic structures of PFD-actin and PFD-CCT complexes suggested a handoff mechanism for the delivery of nonnative actin [16], a higher resolution structure of eukaryotic PFD is necessary to elucidate how PFD recognizes nonnative actin and tubulin. Aikawa et al. have succeeded in obtaining the crystals of human PFD [17], and collected the X-ray diffraction data at 4.7 Å resolution, which was inadequate for determining the crystal structure. The crystallization and preliminary X-ray analysis of one of the β subunits of PFD from *S. cerevisiae* were performed, but the atomic structure could not be determined [18].

Recently, we succeeded in obtaining recombinant CCT from a thermophilic fungus, *Chaetomium thermophilum* (CtCCT) [19]. We obtained the recombinant CtCCT with a relatively high yield, and it exhibited a fairly high thermal stability. In this study, we have expressed, purified, and characterized PFD from *C. thermophilum* (CtPFD). 

## 2. Results

### 2.1. Expression and Purification of the CtPFD

The proteins from *C. thermophilum* were expected to have a high stability. Indeed, several studies on *C. thermophilum* proteins have been reported [20,21]. Recently, we have succeeded in expressing CCT from *C. thermophilum* (CtCCT) in *E. coli* [19]. As expected, CtCCT exhibited a higher structural stability compared with mammalian or yeast CCTs. Then, we decided to express and characterize PFD from *C. thermophilum* (CtPFD) to study the protein-folding mechanism of the PFD-CCT system. Although a draft genome sequence of *C. thermophilum* had been published, the annotation of PFD subunit genes was incomplete. Thus, we cloned and sequenced CtPFD subunit cDNAs from *C. thermophilum* var. *coprophilum* strain NBRC 30,073 (Supplementary Figure S1). The sequence data have been deposited to DDBJ/EMBL/GenBank under the accession numbers of LC388395, LC388396, LC388397, LC388398, LC388399, and LC388400. The amino acid sequences of the CtPFD subunits exhibited significantly high homology scores with other PFD subunits (Supplementary Figure S2).

**Figure 2:**
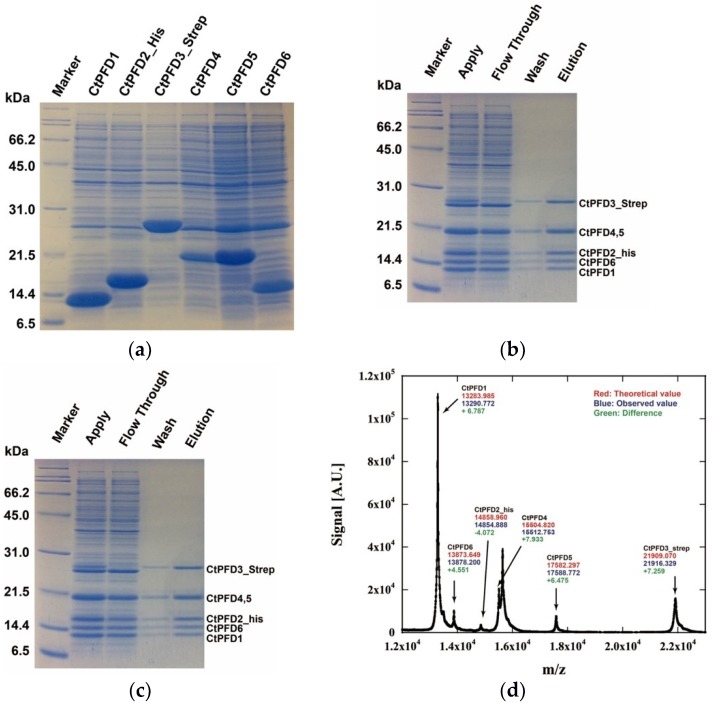
Expression, reconstitution, and purification of *Chaetomium thermophilum* (CtPFD). (**a**) SDS-PAGE analysis of CtPFD subunit expression in *E. coli*. (**b**) SDS-PAGE analysis of the affinity purification of the CtPFD oligomer by StrepTrap HP. (**c**) SDS-PAGE analysis of the affinity purification of the CtPFD oligomer by His-Trap HP. (**d**) MALDI-TOF MS analysis of purified CtPFD. Peaks are annotated with the theoretical molecular mass (red), the observed molecular mass (blue), and the difference value (green).

Each CtPFD subunit was expressed in *E. coli* independently. To obtain the reconstituted hexameric complex by affinity chromatography, a Strep-tag sequence was attached at the C-terminus of PFD3, and the His-tag sequence was attached at the N-terminus of PFD2. All six subunits were highly expressed in *E. coli* (Figure 2a). All purified PFD subunit proteins were denatured with a buffer containing 6 M urea, then mixed in equimolar amounts. Refolding and reconstitution were performed through dilution of urea concentration by dialysis. The reconstituted CtPFD complex was purified by two-step affinity chromatography using the Strep-tag and His-tag (Figure 2b,c). Finally, the CtPFD complex was purified by size-exclusion chromatography. analysis showed five protein bands. One of these corresponded to PFD4 and PFD5. The existence of the six subunits was also confirmed by MALDI-TOF mass spectrometry (Figure 2d). 

**Figure 3:**
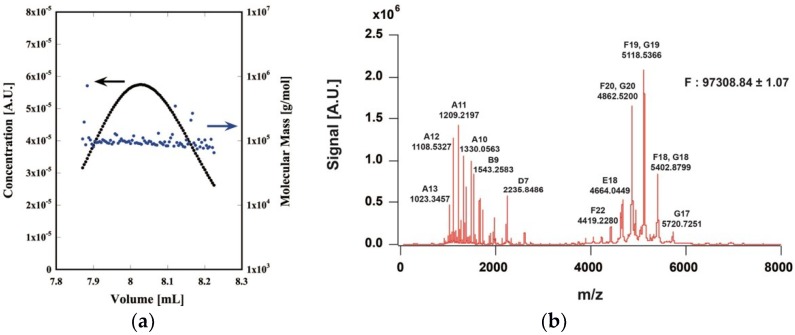
Molecular mass determination of CtPFD. (**a**) SEC-MALS of purified CtPFD. Black, protein concentration (arbitrary units); Blue, molecular mass. (**b**) Native mass spectrometry of CtPFD.

Although the apparent molecular mass of each subunit was partly different from the predicted value, the mass of each subunit was almost the same as the predicted value. The molecular mass of the CtPFD complex was analyzed by chromatography-multiangle light scattering and by native mass spectrometry, and found to be 97.5 and 97.1 kDa, respectively (Figure 3a,b). These are almost the same as the molecular weight estimated from the amino acid sequences (97.2 kDa). Therefore, we concluded that the CtPFD complex was obtained.

### 2.2. Interaction with Substrates

**Figure 4:**
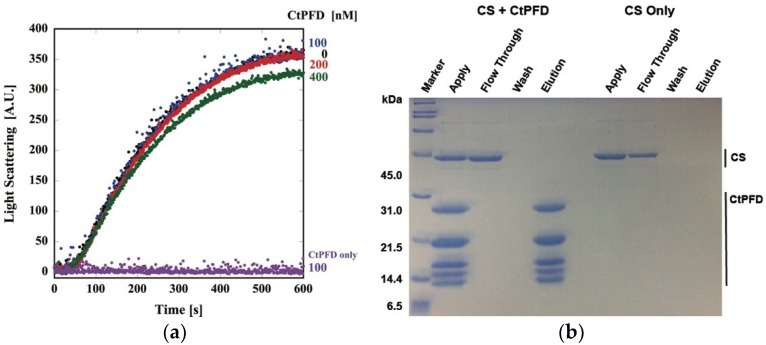
Analysis of CtPFD interaction with denatured CS. (**a**) Effect of CtPFD on the thermal aggregation of CS. (**b**) SDS-PAGE of the pull-down assay for the interaction between StrepTrap HP-immobilized CtPFD and denatured CS.

First, we examined the chaperone activity of CtPFD using unfolded porcine heart citrate synthase (CS) as a nonspecific substrate, as it has been used for archaeal PFD [8]. Thermal aggregation of CS was observed as the increase of light scattering. In contrast, CtPFD did not exhibit any increase of light scattering. Despite the addition of excess CtPFD, CS aggregation was not suppressed (Figure 4a). The interaction between CtPFD and CS was analyzed by a pull-down assay. CS did not coelute with CtPFD (Figure 4b). Therefore, we concluded that CtPFD does not have general chaperone activity.

**Figure 5:**
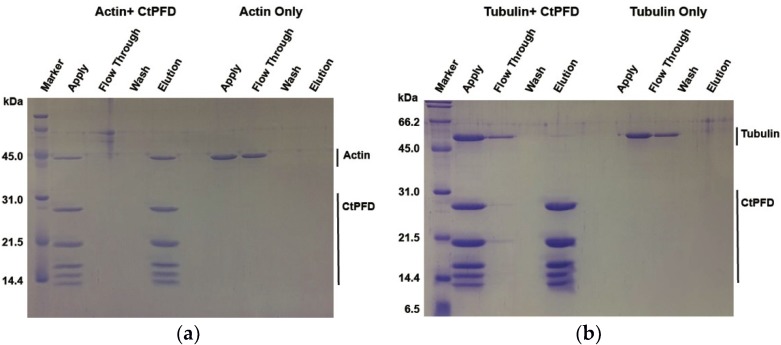
Interaction of CtPFD with substrate proteins. SDS-PAGE of the pull-down assay for the interaction between CtPFD immobilized on StrepTrap HP column with actin (**a**) and tubulin (**b**).

The binding affinities of CtPFD to actin and tubulin were assessed by the pull-down assay. CtPFD was immobilized on a Strep-Tactin column, and acid-denatured actin and tubulin were applied to it. After several washes, CtPFD was eluted by adding 2.5 mM d-desthiobiotin, and the eluted proteins were detected with Coomassie Brilliant Blue-stained SDS-PAGE and by western blots (Figure 5a,b). The results clearly showed that CtPFD bound to denatured actin and tubulin.

### 2.3. Interaction with CtCCT

**Figure 6:**
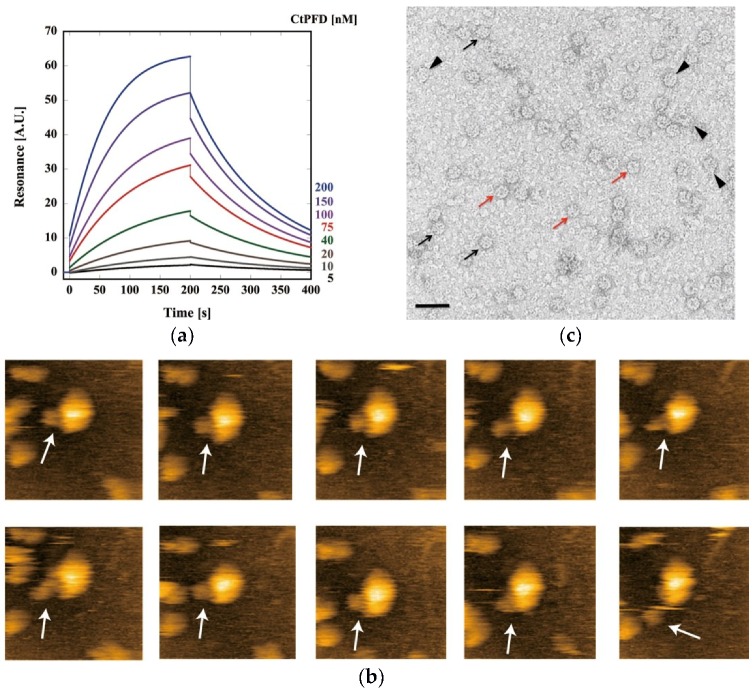
Interaction between CtPFD and CtCCT. (**a**) Sensorgrams of the interactions between CtPFD and CtCCT analyzed with the Biacore T-200 system. (**b**) The time-course of an interaction event between CtPFD and CtCCT observed by HS-AFM. Location of CtPFD is shown with an arrow. (**c**) EM image of CtPFD–CtCCT complexes. Red and black arrows point respectively to end-on and side views of apo-CtCCT, whereas the arrowheads signal the presence of side views of CtPFD-CtCCT complexes. Bar = 500 Å.

The interaction between CtPFD and CCT from *C. thermophilum* (CtCCT) was analyzed by surface plasmon resonance (SPR) using the Biacore T200. When CtPFD was immobilized on the sensor chip by amine coupling, CtCCT produced a concentration-dependent signal (1, 2, 5, 10, 15, 20, 30, 40, 50, 75, 100, 150, 200 nM). Sensorgrams for CtCCT concentrations of 5, 10, 20, 40, 75, 100,150, and 200 nM are shown (Figure 6a). The kinetic parameters *k*_on_, *k*_off_, and *K*_D_ were calculated to be 9.02 × 10^4^, 4.03 × 10^−3^, and 4.47 × 10^−8^, respectively. The *K*_D_ value is similar to that of archaeal counterparts [8]. Although the *K*_D_ value is small, the dissociation rate is high. Thus, the CtPFD–CtCCT complex seems to be unstable. This was confirmed in real time in experiments with high-speed atomic force microscopy (HS-AFM). When CtCCT was immobilized on a mica surface and CtPFD was added, we observed CtPFD–CtCCT complex formation (Figure 6b), and the unstable nature of the CtPFD–CtCCT interaction could be confirmed by electron microscopy, which revealed a low percentage of CtPFD–CtCCT complexes, despite the incubation of CCT and PFD oligomers at a 1:5 ratio (Figure 6c).

### 2.4. Actin Transfer to CtCCT

**Figure 7:**
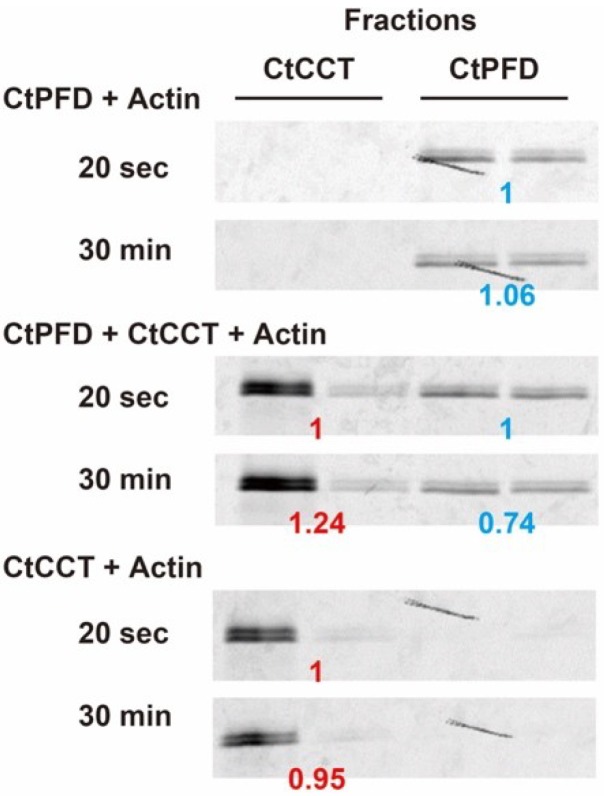
Actin transfer from CtPFD to CtCCT. Fluorescence images of fluorescent actin binding to CtPFD (**top**), to CtPFD and subsequently to CtCCT (**middle**) and to CtCCT (**bottom**). Each band corresponds to different fractions of the experiments. Relative intensities are shown.

Next, we tried to examine actin transfer from CtPFD to CtCCT. Fluorescently labeled actin was denatured in 8 M urea, and renatured in the presence or absence of CtPFD (Figure 7). Fluorescently labeled actin was found only in the fractions of CtPFD (CtPFD + Actin in Figure 7). The fluorescence intensity did not change with incubation time. Consistent with a relatively weak interaction between CtPFD and actin, the fluorescence intensity was weak. The sample was also incubated with CtCCT, and the mixture was separated by size-exclusion chromatography. In this case, the fluorescence was associated with the two fractions that corresponded to CtPFD and CtCCT (CtPFD + CtCCT + Actin in Figure 7). Even in the absence of CtPFD, CtCCT could capture actin (CtCCT + Actin in Figure 7). The fluorescence intensity of the CtCCT fraction increased with time, which did not in the case when fluorescent actin was incubated with CtCCT (CtCCT + Actin in Figure 7). Therefore, we concluded that CtPFD can transfer denatured actin to CtCCT.

### 2.5. Crystallization

**Figure 8:**
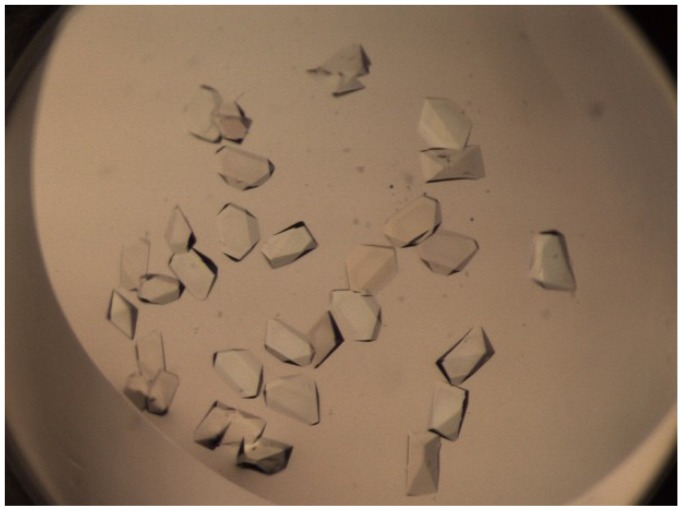
Crystals of CtPFD.

The CtPFD solutions at 15 or 7.5 mg/mL were mixed with 250 kinds of reservoir solutions (JBScreen Classic1-10, Jena Bioscience), and crystals were grown by the vapor diffusion method. The CtPFD crystals were obtained when the chaperone was mixed with 200 mM ammonium di-hydrogen phosphate (Figure 8). However, the best crystals diffracted to only 8 Å resolution, so a detailed structure of CtPFD could not be obtained (Supplementary Figure S3). The diffraction spots can be indexed in an orthorhombic cell with the parameters of a = 154.2, b = 128.9, and c = 78.09 Å. Assuming the presence of eight PFD hexameters in the unit cell, the Matthews coefficient and solvent content were calculated to be 2.0 Å^3^ Da^−1^ and 38.4%, respectively.

## 3. Discussion

PFD and group II chaperonin are recognized to be important players in archaeal and eukaryotic cytosols. However, their reaction mechanism, substrate specificity, and structures are poorly understood, compared with other molecular chaperones. Studies on archaeal PFD and group II chaperonin have advanced using hyperthermophilic archaea. Due to their structural stabilities, they were analyzed by various biophysical methods. The molecular chaperone system of hyperthermophilic archaea is composed of only limited members. Thus, PFD and group II chaperonin are thought to function as the general chaperones. Their structures are relatively simple and recognize the hydrophobic surface of unfolded/denatured proteins. In contrast, eukaryotic PFD and group II chaperonin (CCT/TRiC) require very complex structures consisting of eight and six different subunits, respectively, which makes it difficult to perform detailed biochemical and biophysical studies. Recently, we have succeeded in expression, purification, and characterization of CCT from a thermophilic fungus, *C. thermophilum* (CtCCT) [19]. Its thermal stability enabled us to obtain unprecedented information on CCT. Therefore, we decided to study PFD from *C. thermophilum* (CtPFD). We expressed all subunits of CtPFD in *E. coli* independently, and mixed them in denaturing conditions using urea. Reconstituted CtPFD was obtained by dialysis to remove urea. To obtain the CtPFD complex composed of all subunits, we added tag sequences to two subunits. Following two-step affinity chromatography and gel filtration chromatography, the complete CtPFD composed of six subunits was obtained. 

In the preliminary experiment, we tried to obtain CtPFD with only one tag sequence. Unexpectedly, the protein complex obtained by single affinity chromatography included the incomplete complexes. Recent studies have shown that the prefoldin subunit may function independently. PFD4 expression is a prognostic factor in breast and colorectal tumor and cancer cells [22]. PFD2 was identified as a biomarker of bladder cancer cells [23]. PFD1 was reported to be involved in the progression of colorectal and lung cancer [24,25]. There are reports on noncanonical complexes containing PFD subunits [26]. Mammalian prefoldin subunits two and six form a complex together with UXT, RPB5, WDR92/Monad, PDRG1, and URI [27]. In this complex, it is supposed to adopt a prefoldin-like structure, therefore, it is plausible that PFD may function in various configurations. We examined the chaperone function of CtPFD using CS as the substrate. CtPFD did not interact with denatured CS, and was unable to protect it from thermal aggregation. Thus, it was clearly shown that CtPFD is not the general chaperone. In contrast, we observed the interaction of CtPFD with the canonical substrates, actin and tubulin. CCT/TRiC and PFD were first identified as chaperones for actin and tubulin. Later, it was found that CCT/TRiC facilitates folding of approximately 10% of cytosolic proteins. However, interactive analysis of eukaryotic PFD is poorly understood. We will analyze the substrates of PFD using this system. We have succeeded in obtaining the crystal of CtPFD. Despite our expectation, it yielded poor diffractions. In the crystal structures of archaeal PFDs, the termini of tentacles were very flexible, and impaired the diffractions [9,10]. Thus, we are planning to construct CtPFDs for X-ray crystallography.

## 4. Materials and Methods

### 4.1. Bacterial Strains, Fungi, Proteins, and Reagents

The *Escherichia coli* strains used in this study were DH5α for plasmid propagation, and BL21 (DE3) star pRARE and Rossetta2 (DE3) pLysS (Invitrogen, Carlsbad, CA, USA) for protein expression. *C. thermophilum* was obtained from NBRC (Biological Resource Center, National Institute of Technology and Evaluation, Tokyo, Japan). The protein concentrations were determined using the Bio-Rad protein assay (Bio-Rad, Hercules, CA, USA) with bovine serum albumin as a standard. Total RNA of *C. thermophilum* was obtained using the NucleoSpin RNA Plant (Takara Bio, Shiga, Japan), and cDNA was synthesized by the cDNA Synthesis Kit (Takara Bio). KOD-Plus-Neo DNA polymerase used for gene amplification and restriction endonucleases were obtained from TOYOBO (Osaka, Japan) and New England Biolabs Japan (Tokyo, Japan), respectively. Nucleotides and other reagents were purchased from Wako Pure Chemical Industries (Osaka, Japan) or Sigma-Aldrich Japan (Tokyo, Japan). Actin was extracted from an acetone-dried powder made from chicken white breast meat, and was purified by ultracentrifugation, as previously described for rabbits [28]. Tubulin was purified from porcine brain with polymerization and depolymerization cycling [29]. CtCCT was expressed and purified as described previously [19].

### 4.2. Cloning, Expression, and Purification of CtPFD

The cDNAs encoding CtPFD subunits were obtained by RT-PCR from mRNA isolated from *C. thermophilum*. The fungus was cultured in a potato–carrot medium at 40 °C. The cDNA of CtPFD subunits was obtained by PCR amplification from cDNA using the primer pairs shown in Supplementary Table S1. The amplified genes were digested by restriction enzymes, cloned into pMD20 (TAKARA), and their sequences were verified. CtPFD subunit cDNAs were cloned into pET23b (Merck Millipore, Billerica, MA, USA) between the NdeI and BamHI sites (except for CtPFD3) or the NdeI and XhoI sites (CtPFD3). Among six subunits, the Strep-tag sequence was attached at the C-terminus of an α type subunit, PFD3 (PFD3_Strep), and the 6× Histidine-tag sequence was attached at the N-terminus of a β type subunit, PFD2 (PFD2_His). *E. coli* BL21 (DE3) star pRARE or Rossetta2 (DE3) pLysS cells were transformed with the expression plasmids and cultured for approximately 24 h at 37 °C in LB medium containing ampicillin and chloramphenicol. After ultrasonic homogenization, proteins were analyzed by SDS-PAGE.

Total protein of recombinant *E. coli* cells expressing 6 different subunits were mixed in the presence of 6 M urea, and refolding of subunits and reassembly of CtPFD were performed by a gradual decrease in urea concentration. Reconstituted CtPFD was purified by affinity chromatography using a StrepTrap HP column (GE Healthcare, Buckinghamshire, UK) for the Strep-tag, and a His-Trap HP column (GE Healthcare) for the 6× His-tag. Finally, the CtPFD was purified by size-exclusion chromatography using a HiLoad 26/600 Superdex 200 pg column (GE Healthcare). 

### 4.3. Size Exclusion Chromatography-Multiangle Light Scattering (SEC-MALS)

The purified CtPFD were analyzed by SEC-MALS on a WTC-100S5 column (Wyatt Technology, Santa Barbara, CA, USA) equipped with a multiangle light-scattering detector (MINI DAWN, Wyatt Technology) and a differential refractive index detector (Shodex RI-101, Showa Denko, Tokyo, Japan) in a PU-980i HPLC system (JASCO). A 100-µL aliquot was injected into the column and eluted with the buffer (50 mM Tris-HCl pH 8.0, 5 mM MgCl2, 100 mM KCl) at 1.0 mL/min. The molecular weight and protein concentration were determined according to the instruction manual (Wyatt Technology).

### 4.4. Native Mass Spectrometry

Native mass spectrometry was performed as described previously [30]. Ten-micromolar prefoldin was buffer-exchanged into a 150 mM ammonium acetate buffer, pH 8.0, at 4 °C, by passing the sample through a MicroBioSpin-6 column (Bio-Rad, Hercules, CA, USA). The buffer-exchanged sample was immediately analyzed by nanoflow electrospray-ionization mass spectrometry using gold-coated glass capillaries made in house (approximately 2–5 µL sample loaded per analysis). The spectrum was recorded on a SYNAPT G2-Si HDMS mass spectrometer (Waters, Milford, MA, USA) in a positive ionization mode at 1.33 kV with a 150-V sampling cone voltage and source offset voltage, 0 V trap and transfer collision energy, and 5 mL/min trap gas flow. The spectrum was calibrated using 1 mg/mL cesium iodide and analyzed using the MassLynx software (Waters). 

### 4.5. Protection of Thermal Aggregation of CS

The thermal aggregation of CS was monitored by measuring light scattering at 500 nm with a FP-6500 spectrofluorometer (JASCO, Tokyo, Japan) for 20 min at 50 °C. Native CS was diluted to a final concentration of 100 nM (as a monomer) with or without CtPFD. As a control, the light scattering of 100 nM CtPFD without CS was also measured. The reaction mixtures were pre-incubated for 10 min at 50 °C and continuously stirred throughout the measurement.

### 4.6. Pull-Down Assay

For the pull-down assay, 14 µM CS, 100 µM actin (Sigma-Aldrich, Japan) and 50 µM tubulin were denatured in a unfolding buffer (6 M guanidine hydrochloride, 50 mM Tris-HCl pH 8.0, 100 mM KCl, 5 mM MgCl_2_) at room temperature for 1 h. The unfolded CS, actin, or tubulin were diluted into a binding buffer (50 mM Tris-HCl pH 8.0, 100 mM KCl, 5 mM MgCl_2_) and immediately applied to the StrepTrapHP column (GE Healthcare), which was bound with CtPFD in advance. The bound proteins were eluted with the elution buffer (binding buffer with 2.5 mM d-desthiobiotin). Total proteins in the elution fraction were precipitated by the addition of trichloroacetic acid. The proteins were separated on 10% SDS gels and were transferred onto a 0.22-µm polyvinylidene difluoride membrane (Millipore) using a transfer buffer (25 mM Tris, 200 mM glycine, 5% methanol). The primary antibody used for actin was the anti-β actin antibody (mAbcam 8224, Abcam Co. Ltd., Tokyo, Japan), and the antibody for tubulin was the monoclonal anti-α-tubulin antibody (T5168, Sigma-Aldrich, Japan). The secondary antibody was an anti-mouse IgG horseradish-peroxidase-linked whole antibody (NA931V, GE Healthcare). The membranes were visualized using the Western BLoT Chemiluminescence HRP Substrate (TaKaRa Bio) and scanned with the Typhoon 8600 (GE Healthcare).

### 4.7. Analysis of CtPFD–CtCCT Interaction by SPR

SPR experiments were performed using the Biacore T200 (GE Healthcare Life Sciences). CtPFD and BSA were immobilized (BSA:Δ3500 RUs, CtPFD:Δ1000 RUs) on the measurement path and BSA (Δ4500) was immobilized on the reference path. CtCCT at different concentrations was applied on these paths. The *K*_D_ was obtained by fitting to the SPR signal data with a recursive least-squares algorithm by KaleidaGraph (Synergy Software, Reading, PA, USA) using the following equation:

*R*_eq_ = *R*_max_*C*/(*C* + *K*_D_)

where *R*_eq_ represents the resonance units (RU) at equilibrium, *R*_max_ is the resonance signal at saturation, and *C* is the injected CtCCT concentration. *R*_eq_ values were obtained by fitting to the sensorgrams using the Langmuir binding model with the BIAevaluation software (GE Healthcare). 

### 4.8. HS-AFM Observation

We obtained single-molecule images of CtPFD and CtCCT using a home-built high-speed atomic force microscopy (HS-AFM) apparatus. The interaction between CtPFD and CtCCT was observed in real time using HS-AFM. Forty nanomolar CtCCT (containing 1% glutaraldehyde to fix the structure of CtCCT) was immobilized on a mica surface, and a droplet containing 20 nM CtPFD was applied. We used a scan area of 40 × 40 nm^2^ with 60 × 60 pixels or 100 × 100 nm^2^ with 100 × 100 pixels at a scan rate of 0.1 s/frame or 0.5 s/frame. A low-pass filter was applied to images to remove noise. The cantilevers were designed for high-speed scanning (spring constants ~0.1 N/m, resonant frequency 0.8–1 MHz, and quality factor ~2 in water, respectively) and operated in tapping mode. For HS-AFM imaging, the free oscillation amplitude was ~1 nm, and the set-point amplitude was approximately 80% of the free oscillation amplitude.

### 4.9. Transmission Electron Microscopy

Aliquots of a solution containing CtPFD and CtCCT were applied to 300 mesh grids (Maxtaform Cu/Rh HR26) coated with a thin (~8 nm) carbon layer and glow-discharged for 15 s. The grids were then stained (1 min) with 2% uranyl acetate and air-dried before transmission EM analysis. Images were acquired using an FEI Tecnai G2 FEG200 transmission electron microscope operated at 200 kV and equipped with a 4k FEI Eagle CCD camera. Images were recorded at a sampling rate of 3.65 Å/px.

### 4.10. Actin Delivery Assay

Actin was fluorescently labeled using Alexa Fluor 488 NHS ester (Thermo Fisher Scientific, Waltham, MA, USA), and was denatured in the presence of 8 M urea on ice for 1 h. The denatured labeled actin was mixed with the 4-fold molar excess of CtPFD in a seven-fold volume excess dilution buffer (20 mM HEPES pH 8.0, 75 mM KCl, 1 mM DTT) on ice for 1 h, and was then mixed with equimolar CtCCT. After 20 s or 30 min, the aliquot of the mixture was applied onto a TSK gel G3000SWXL column (TOSOH, Tokyo, Japan). The fractions were analyzed by SDS-PAGE and fluorescence detection using Typhoon 8600. The fluorescence intensities of the CtCCT and CtPFD fractions were measured, and the relative intensities between 30 min and 20 s were calculated. The control experiments were performed without an addition of CtPFD or CtCCT (CtPFD + actin, CtCCT + actin).

### 4.11. Crystallization and X-ray Data Collection

CtPFD (15 or 7.5 mg/mL) was mixed with 250 kinds of reservoir solutions (JBScreen Classic1-10, Jena Bioscience) and crystals were grown by the vapor diffusion method. The CtPFD crystals were obtained when CtPFD was mixed with 200 mM ammonium di-hydrogen phosphate. The crystals were soaked in a reservoir solution supplemented with 20% (*w*/*v*) PEG 4000, and flash-cooled in a nitrogen-gas stream at 95 K. The X-ray diffraction data were collected on beamline BL1A at the Photon Factory.

## Supplementary Materials

Supplementary Materials can be found at http://www.mdpi.com/1422-0067/19/8/2452/s1.

## Abbreviations

CCT	Chaperonin containing TCP-1
PFD	Prefoldin
TRiC	TCP-1 ring complex
CtCCD	CCT from *Chaetomium thermophilum*
CtPFD	PFD from *Chaetomium thermophilum*
PFD1	Subunit 1 of CtPFD
PFD2	Subunit 2 of CtPFD
PFD3	Subunit 3 of CtPFD
PFD4	Subunit 4 of CtPFD
PFD5	Subunit 5 of CtPFD
PFD6	Subunit 6 of CtPFD
CS	Porcine heart citrate synthase
SPR	Surface plasmon resonance
HS-AFM	High-speed atomic force microscopy
