# Supplementary material for: Expression, Functional Characterization, and Preliminary Crystallization of the Cochaperone Prefoldin from the Thermophilic Fungus Chaetomium thermophilum
Source: Int J Mol Sci. 2018 Aug 19;19(8):2452. doi: 10.3390/ijms19082452 (PMC6121465; doi:10.3390/ijms19082452)
Supplement: Supplementary file 1 [file ijms-19-02452-s001.pdf]

Supplementary Fig. S1 Nucleotide and amino acid sequences of CtPFD subunits.

CtPFD1
121 a.a. MW=13423.81

```
ATGTCACTAAGCACAGAGGCGCTACAAAAGCTTGTGCGGGAGATTGAGTCCCAGGCGATTGCGGCACAGCAGCAGATCAGCCTGGTGAGG
 M  S  L  S  T  E  A  L  Q  K  L  V  R  E  I  E  S  Q  A  I  A  A  Q  Q  Q  I  S  L  V  R

ACACAAATCGCAGCCAAGCAGCGGGAGATGCGGCTGGCGCAGTTGACGCGCAGCGAAATCTCCGCACTGCCCAGCGATACGGACGTATAT
 T  Q  I  A  A  K  Q  R  E  M  R  L  A  Q  L  T  R  S  E  I  S  A  L  P  S  D  T  D  V  Y

GAAGGTGTTGGGAAGATGTTCGTGGCGGTGCCGGTGCCGGCGATGCAGGATAAGCTGGGGGAGCAGATCAAGACTCTAGAGGGCGAGGTG
 E  G  V  G  K  M  F  V  A  V  P  V  P  A  M  Q  D  K  L  G  E  Q  I  K  T  L  E  G  E  V

GAGACGATGAACAAGAGGCTGCACTACCTGGAGACAACTGCGAAGAACAGCCAGGAGCACATCGAGAAGATTATCAAGGGCGGTGGTCAG
 E  T  M  N  K  R  L  H  Y  L  E  T  T  A  K  N  S  Q  E  H  I  E  K  I  I  K  G  G  G  Q

TCATAA
 S  *
```

CtPFD2
117 a.a. MW=13449.85

```
ATGGCGACACAGTCTGCAGCAGCAAAGAGGCAGCAAGACCTCCAGCTGCAGTATACCACTTATAAGAACACCCTGCAGCAAATTGCCCAG
 M  A  T  Q  S  A  A  A  K  R  Q  Q  D  L  Q  L  Q  Y  T  T  Y  K  N  T  L  Q  Q  I  A  Q

AAGATTGGAGATGTCGAGCAGGAGGCTGAGGAGCACAAGCTTGTTCTTGAGACTTTGGAGCCTCTCCCAGGTGACCGCAAATGCTTCCGT
 K  I  G  D  V  E  Q  E  A  E  E  H  K  L  V  L  E  T  L  E  P  L  P  G  D  R  K  C  F  R

ATGATCAATGGCGTCTTGGTGGAAAGAACCGTCCAGGATGTGATTCCTGCTCTTCGGACCAACGCTGAAGGGCTGAAGAAGGTCCTCGAA
 M  I  N  G  V  L  V  E  R  T  V  Q  D  V  I  P  A  L  R  T  N  A  E  G  L  K  K  V  L  E

GATCTTGTAAAACAATACAAGGCGAAACAGGATGAACTTGAGCAGTGGAAGAAGAAAAACAACGTTCAGGTCGTGCAGTCATGA
 D  L  V  K  Q  Y  K  A  K  Q  D  E  L  E  Q  W  K  K  K  N  N  V  Q  V  V  Q  S  *
```

CtPFD3
180 a.a. MW=20558.89

```
ATGGCCAGTCAAGCAAAAGAAGCGGCGACAAAGGACGCTACCCCCTCGAACCCCCGCGGCATTCCCTATGCGCCGTTCGTCGACAAGGTC
 M  A  S  Q  A  K  E  A  A  T  K  D  A  T  P  S  N  P  R  G  I  P  Y  A  P  F  V  D  K  V

GAGGACTATGTGTCGTCACGGGACGATGTCGAGCCGACGCTGCGGCGATTCCAAGAGCTGATCGCGAAGTATCAGTTCATGGAGCAGAAC
 E  D  Y  V  S  S  R  D  D  V  E  P  T  L  R  R  F  Q  E  L  I  A  K  Y  Q  F  M  E  Q  N

CTGCAGCGGAGGGTAGCCGGTCTGAAGGATAAGATGCCCGACATCCAAAAGACCTTGGATACGGTGCGGTTCTTGAAGCTCAGGACGTCC
 L  Q  R  R  V  A  G  L  K  D  K  M  P  D  I  Q  K  T  L  D  T  V  R  F  L  K  L  R  T  S

GAGTCGGATCCGATCGAGACAACCTTTGAGCTGAACGACACGCTCTATGCGAAGGCCGAGATCCCGCCGACGGACGAGGTCTATCTCTGG
 E  S  D  P  I  E  T  T  F  E  L  N  D  T  L  Y  A  K  A  E  I  P  P  T  D  E  V  Y  L  W

CTTGGTGCCAACGTCATGCTAGCGTATCCTCTCGACGAAGCAGAGCAGCTCCTGGATTCGAAACTCAAGGCGGCAAAGCAGAGTCTGGCT
 L  G  A  N  V  M  L  A  Y  P  L  D  E  A  E  Q  L  L  D  S  K  L  K  A  A  K  Q  S  L  A

AACTGCGAGGAGGACCTGGACTTTCTGCGCGAGCAGATCACGACCATGGAGGTCGCCATCGCGAGAGTGTACAACTGGGACGTCAGCTCA
 N  C  E  E  D  L  D  F  L  R  E  Q  I  T  T  M  E  V  A  I  A  R  V  Y  N  W  D  V  S  S

TAA
 *
```

CtPFD4
134 a.a. MW=15645.89

```
ATGAACATAAGTCGGCGAATGGAGGAGGAGGAGGCTATAGGCGAGGATGTCGAGGTACGCCGCGAGGACCAGGACAAGATCAACAGGTTC
 M  N  I  S  R  R  M  E  E  E  E  A  I  G  E  D  V  E  V  R  R  E  D  Q  D  K  I  N  R  F

AGCCGTCTGCATGGCCACGAATTGGCGATTGAGGAGGAGCTGAAGGCGAAGCAGAAGGAGAAGGAAGAGTTGGACGATATCACAACGGAG
 S  R  L  H  G  H  E  L  A  I  E  E  E  L  K  A  K  Q  K  E  K  E  E  L  D  D  I  T  T  E

TTGGAGCTTGCCGACGAAGACGAGCTTGTCCCATACAAAGTCGGCGACGCATTCTTCCACATTCCCCTCCCCCAAGCCCAAGAGATGCTC
 L  E  L  A  D  E  D  E  L  V  P  Y  K  V  G  D  A  F  F  H  I  P  L  P  Q  A  Q  E  M  L

GGTATTTCGACGGCGAAGCTCGAGGAGGAAATCTCACAACTTGAGGAGAAGCTGAGCAATATCCGCGATGAGATGTCACAACTAAAGGTG
 G  I  S  T  A  K  L  E  E  E  I  S  Q  L  E  E  K  L  S  N  I  R  D  E  M  S  Q  L  K  V

GAGTTGTACGCAAGATTTGGCAAGACCATCAATCTCGAGACATGA
 E  L  Y  A  R  F  G  K  T  I  N  L  E  T  *
```

CtPFD5
165 a.a. MW=17724.67

```
ATGGCCAGCCGCAGCGGTCAAGATAAACAAGGACAAGGAGAGGCAGTCGATCTTGCATCCCTCAGCGTACAACAACTCAGTACAGTGAAG
 M  A  S  R  S  G  Q  D  K  Q  G  Q  G  E  A  V  D  L  A  S  L  S  V  Q  Q  L  S  T  V  K

AAGCAGCTTGACGGAGAAGTCGAGCACCTATCAACGTCGTACGCGCAGTTGGTGGCCGCTCAGGGGAAGTTTAAAGAATGTCTACGCATT
 K  Q  L  D  G  E  V  E  H  L  S  T  S  Y  A  Q  L  V  A  A  Q  G  K  F  K  E  C  L  R  I

GTCAAGGACAGAGCATCAACGCTTGAGAAGGATAAAACCGTTCTCGTTCCCCTTACCAACTCCCTCTACGTCAAGGGAAAGCTGTCCGAC
 V  K  D  R  A  S  T  L  E  K  D  K  T  V  L  V  P  L  T  N  S  L  Y  V  K  G  K  L  S  D

CCGGAACACGTGCTCGTTGATGTCGGAACGGGCTTCTACGTGGAAAAGGACACGACCAGCGCAGCTGAATTTTACGAAGGCAAGATCAAG
 P  E  H  V  L  V  D  V  G  T  G  F  Y  V  E  K  D  T  T  S  A  A  E  F  Y  E  G  K  I  K

GAGCTAGCGACCAATATCCAGGGACTGGAGGGCATTGTGCAGGCAAAAACAGCAAATCTCCGAGTTGTGGAAGAAGTCCTTCGGCAAAAG
 E  L  A  T  N  I  Q  G  L  E  G  I  V  Q  A  K  T  A  N  L  R  V  V  E  E  V  L  R  Q  K

GTACTCGCTTCAAACGCTGCTGGAACGGGAGCAAAGCAATCTTCATGA
 V  L  A  S  N  A  A  G  T  G  A  K  Q  S  S  *
```

CtPFD6
124 a.a. MW=14013.59

```
ATGGCTGAAATCCAGGCCCGCCTTCAAGCCCTCTCTGAGGAGTTCAGCAAGCTCCAACAAGATCTCCAAGATGCTCTGCAATCAAGACAG
 M  A  E  I  Q  A  R  L  Q  A  L  S  E  E  F  S  K  L  Q  Q  D  L  Q  D  A  L  Q  S  R  Q

AAGCTCGAGGCGCAGAAGCAAGAAAACCTTGGGGTCCAAAAGGAGTTCAAGAAGCTCAAGGAGGGCGAAAACATCTACAAACTGATCGGC
 K  L  E  A  Q  K  Q  E  N  L  G  V  Q  K  E  F  K  K  L  K  E  G  E  N  I  Y  K  L  I  G

CCTGTTCTTCTCAAGCAAGACAAGGTTGATGCAGAGAGCATAGTCAATGGTCGTCTAGACTTTATTGACAAGGAAATCAACCGAATAGAA
 P  V  L  L  K  Q  D  K  V  D  A  E  S  I  V  N  G  R  L  D  F  I  D  K  E  I  N  R  I  E

ACTCTCATCAAGGAACTCCAGGGCAAGATCGAGAAAAAGAAAGGGGAGATTATCCAGGTGCAAAGTGGTTTGCAAGCAGCGGCAGTTCAA
 T  L  I  K  E  L  Q  G  K  I  E  K  K  K  G  E  I  I  Q  V  Q  S  G  L  Q  A  A  A  V  Q

GCCGTCAAGGCATGA
 A  V  K  A  *
```

Supplementary Fig. S2 Amino acid sequence alignments of Prefoldin subunits

```
CtPFD1      MSLS-TEALQKLVREIESQAIAAQQQISLVRTQIAAKQREMRLAQLTRSEISALPSDTDV
ScPFD1      --------MSQIAQEMTVSLRNARTQLDMVNQQLAYLDRQEKLAELTKKELESYPTD-KV
HsPFD1      MAAPVDLELKKAFTELQAKVIDTQQKVKLADIQIEQLNRTKKHAHLTDTEIMTLVDETNM
EnPFD1      MSIP-NEALQKLLQEIESRVITSQQQIGITKAQMTSKNRDIRMLELTSKELSSLPADTNV
                    :.:   *:      :: :: :.  *:   :*  :  .** .*: :   : .:

CtPFD1      YEGVGKMFVAVPVPAMQDKLGEQIKTLEGEVETMNKRLHYLETTAKNSQEHIEKIIKGGG
ScPFD1      WRSCGKSFILQDKSKYVNDLSHDETVLLDQRKTLKIKKNYLETTVEKTIDNLKALMKN--
HsPFD1      YEGVGRMFILQSKEAIHSQLLEKQKIAEEKIKELEQKKSYLERSVKEAEDNIREMLMARR
EnPFD1      YEGVGKMFVAVPRATVDKRISTETGELKGEIENLEKRMNYLEMTFKNSRENLEAILKSGR
            :.. *: *:        . :  .      : : :: :  *** : ::: :::. ::

CtPFD1      QS
ScPFD1      --
HsPFD1      --
EnPFD1      A-
```

Ct: *Chaetomium thermophilum*
Sc: *Saccharomyces cerevisiae*
Hs: *Homo sapiens*
En: *Emericella nidulans*

```
CtPFD2          ------------MATQSAAAKRQQDLQLQYTTYKNTLQQIAQKIGDVEQEAEEHKLVLET
ScPFD2          ------------------MEQRNNVFQAKYNEYKQILEELQTKIIELGHDKDEHTIVIKT
HsPFD2          MAENSGRAGKSSGSGAGKGAVSAEQVIAGFNRLRQEQRGLASKAAELEMELNEHSLVIDT
EnPFD2          -----------MASQPQIAPKKQQELQAQYTNFKNTLQQLAQKIGDIEQEAEEHKLVIDT
                                       : .   :.  ::  . :  *  ::  : :**.:*:.*

CtPFD2          LEPLPGDRKCFRMINGVLVERTVQDVIPALRTNAEGLKKVLEDLVKQYKAKQDELEQWKK
ScPFD2          LKDAEPTRKCYRMIGGALVESDVQTSLPILETKKENIEGTISKMKETLIQTAKEFEKWKK
HsPFD2          LKEVDETRKCYRMVGGVLVERTVKEVLPALENNKEQIQKIIETLTQQLQAKGKELNEFRE
EnPFD2          LEPLPQDRTCFRMVNGVLVERTVADVLPTLKTNSDGLKQVLEDMLKQYKSKQSELDNWKK
                *:     *.*:**:.*.***  *   :* *..: : ::  :. : :    . .*::::::

CtPFD2          KNNVQVVQS
ScPFD2          DNKIQVVKN
HsPFD2          ---------
EnPFD2          KNNIQVVQP
```

```
CtPFD3      MA----SQAKEAATKDATPSNPRGIPYAPFVDKVEDYVSSRDD--VEPTLRRFQELIAKY
ScPFD3      ----------MDTLFNSTEKNARGIPQAPFIENVNEIIKDPSD--FELCFNKFQERLSKY
HsPFD3      MAAVKDSCGKGEMATGNGRRLHLGIPEAVFVEDVDSFMKQPGNETADTVLKKLDEQYQKY
EnPFD3      --------MADKKPSDPTQTNPRGIPVAPFVDNVSDYVSSRAD--VEPTMRSFQEMISKY
                           .       *** * *::.*.. :..  :   :  :. ::*   **

CtPFD3      QFMEQNLQRRVAGLKDKMPDIQKTLDTVRFLKLRT----SESDPIETTFELNDTLYAKAE
ScPFD3      KFMQESKLATIKQLKTRIPDLENTLKICQSLRNHSDEGDESDEPILLHYQLNDTLYTKAQ
HsPFD3      KFMELNLAQKKRRLKGQIPEIKQTLEILKYMQKKK----ESTNSMETRFLLADNLYCKAS
EnPFD3      QFMEVNTSRRAAGLREKIPDIKKTLEMVTFLKARKE--SSSATPLETNFELNDTLYARAT
            :**: .       *: ::*::::**.    :: :.    ..   :   : * *.** :*

CtPFD3      IPP-----TDEVYLWLGANVMLAYPLDEAEQLLDSKLKAAKQSLANCEEDLDFLREQITT
ScPFD3      VDIPEDRADLKVGLWLGADVMLEYPIDEAIELLKKKLADSEQSLTVSTEDVEFLRENITT
HsPFD3      VPP-----TDKMCLWLGANVMLEYDIDEAQALLEKNLSTATKNLDSLEEDLDFLRDQFTT
EnPFD3      VDPAH---TEEVYLWLGANVMLAYPIAEAQAMLEDKLAAAEQSLANCDEDLEFLREQITT
            :         :: *****:*** * : **  :*..:*  : :.*    **::***:::**

CtPFD3      MEVAIARVYNWDVSS----------------
ScPFD3      MEVNCARLYNWDVQRRQDLKQAQEGTKNLKI
HsPFD3      TEVNMARVYNWDVKRRNKDDSTKNKA-----
EnPFD3      LEVATARVYNWDVVQRRKERAEGKEETS---
             **  **:*****
```

```
CtPFD4      ----MNISRRMEEEEAIGEDVEVRREDQDKINRFSRLHGHELAIEEELKAKQKEKEELDD
ScPFD4      ---------MELLPQGQRNNTQVTFEDQQKINEFSKLIMRKDAIAQELSLQREEKEYLDD
HsPFD4      --------MAATMKKAAAEDVNVTFEDQQKINKFARNTSRITELKEEIEVKKKQLQNLED
EnPFD4      MMNHRMLSKEDEAAIGNSEDTEVRREDQEKINRFSRLHQRETVLEEKLKGKQKDKEDLEE
                           .  ::.:*  ***:***.*::   :   : :::. :::: : *::

CtPFD4      ITTELELADEDE-LVPYKVGDAFFHIPLPQAQEMLGISTAKLEEEISQLEEKLSNIRDEM
ScPFD4      VSLEIELIDEDE-PVQYKVGDLFIFMKQSKVTAQLEKDAERLDNKIETLEDKQRDIDSRL
HsPFD4      ACDDIMLADDDCLMIPYQIGDVFISHSQEETQEMLEEAKKNLQEEIDALESRVESIQRVL
EnPFD4      ISTELELADEDE-LIPYKIGDSFVHLPLEEAQTLLASSTEQIDSEVAKLEETLSDLRDEM
               :: * *:*   : *::** *.     :.   *     .::.::  **.   .:   :

CtPFD4      SQLKVELYARFGKTINLET---
ScPFD4      DALKAILYAKFGDNINLER---
HsPFD4      ADLKVQLYAKFGSNINLEADES
EnPFD4      QQLKVALYARFGRSINLET---
              **. ***:** .****
```

```
CtPFD5      --MASRSGQDKQGQGEAVDLASLSVQQLSTVKKQLDGEVEHLSTSYAQLVAAQGKFK---
ScPFD5      ------------MSSQKIDLTKLNPEQLNAVKQQFDQELQHFTQSLQALTMAKGKFT---
HsPFD5      -------------MAQSINITELNLPQLEMLKNQLDQEVEFLSTSIAQLKVVQTKYVEAK
EnPFD5      MPPQNTPASDSDAPPGAVNISSLSTPQLRALQTRLSTELEHLTSSHAKLRAAQSRFR---
                             ::::.*.  **  :: ::. *::.:: *   *  .: ::

CtPFD5      ECLRIVKDRAS-----TLEKDKTVLVPLTNSLYVKGKLSDPEHVLVDVGTGFYVEKDTTS
ScPFD5      ECIDDIKTVS-----QAGNEGQKLLVPASASLYIPGKIVDNKKFMVDIGTGYYVEKSAEA
HsPFD5      DCLNVLNKS---------NEGKELLVPLTSSMYVPGKLHDVEHVLIDVGTGYYVEKTAED
EnPFD5      DCVRSINEGVIGSAKKGTEGKDEILVPLTSSLYVKGRLTDREKVLVDVGTGYYVEKTAAK
            :*:  ::           :  . :*** : *:*: *:: * ::.::*:***:**** :

CtPFD5      AAEFYEGKIKELATNIQGLEGIVQAKTANLRVVEEVLRQKVLASNAAGTGAKQSS-----
ScPFD5      AIAFYQKKVDKLNKESVQIQDIIKEKTQYSLSIEAQIRQAAIRQHEAMSKQQQQQQKKES
HsPFD5      AKDFFKRKIDFLTKQMEKIQPALQEKHAMKQAVMEMMSQKIQQLTALGAAQATAKA----
EnPFD5      AIEFYEQKVKELETNLTELEKLVQTKSSQQRLFEDALRQKLMSEGAASSAQAAAAG----
            *  *:: *:. * .:   ::  :: *      .   : *         :

CtPFD5      ---
ScPFD5      STA
HsPFD5      ---
EnPFD5      ---
```

```
CtPFD6      -MAEIQARLQALSEEFSKLQQDLQDALQSRQKLEAQKQENLGVQKEFKKLKEGENIYKLI
ScPFD6      --------MSELGAKYQQLQNELEEFIVARQKLETQLQENKIVNEEFDQLEEDTPVYKLT
HsPFD6      MAELIQKKLQGEVEKYQQLQKDLSKSMSGRQKLEAQLTENNIVKEELALLDGSNVVFKLL
EnPFD6      -MADPQKQMQALSDEYQALQTELDGLVDARQKLESQQQENKSVQAEFNSLDDDANIFKLI
                    :.    ::. ** :*.  : .*****:*  **  *: *:  *. .  ::**

CtPFD6      GPVLLKQDKVDAESIVNGRLDFIDKEINRIETLIKELQGKIEKKKGEIIQVQSGLQAAAV
ScPFD6      GNVLLPVEQSEARTNVDKRLEFIETEITRCEKNIRDKQEELEKMRSELIKLNNTAASTGP
HsPFD6      GPVLVKQELGEARATVGKRLDYITAEIKRYESQLRDLERQSEQQRETLAQLQQEFQRAQA
EnPFD6      GPVLLKQDKTEALMAVNGRLEFIEKEIQRIEGQIKENQDKSDKKRAEIVQYQSQIQQQAA
            * **:  : :*    *. **::*  ** * *  ::: : : :: :  : : :.

CtPFD6      QAVKA----
ScPFD6      GR-------
HsPFD6      AKAGAPGKA
EnPFD6      AAAASA---
```

Supplementary Fig. S3 X-ray diffraction pattern from a crystal of CtPFD.

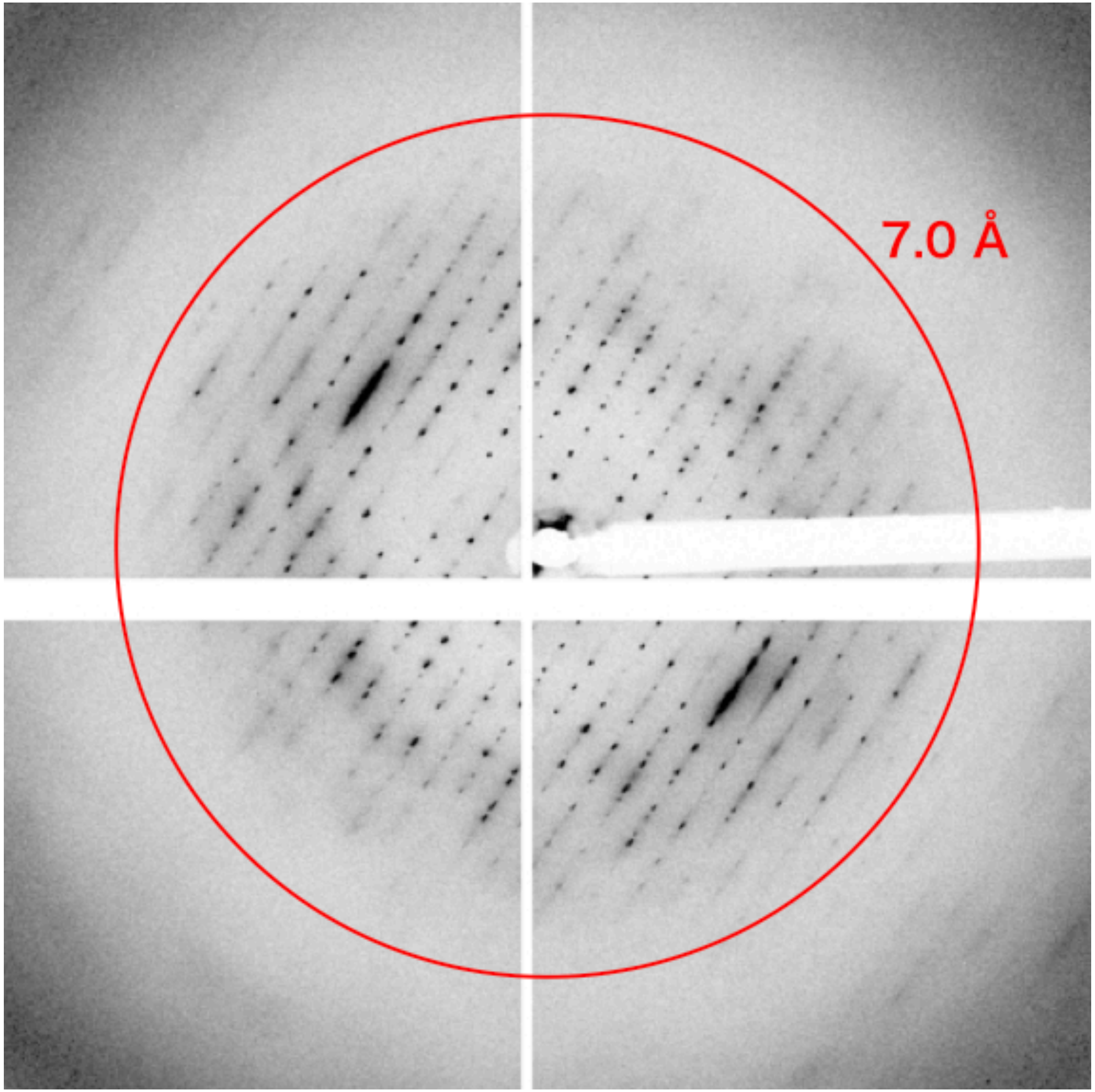

Supplementary Table S1 Primers, vectors, host strains for expression of PFD subunits

PFD1
Forward primer: GGAATTCCATATGTCACTAAGCACAGAGGCGC
Reverse primer: CGGGATCCATTATGACTGACCACCGCCCTT
Expression vector: pET-23b
Expression host: *E coli.* BL21 star (DE3) pRARE
Complete amino-acid sequence of the construct produced:
MSLSTEALQKLVREIESQAIAAQQQISLVRTQIAAKQREMRLAQLTRSEISALPSDTDVYEGVGKMFVAVPVPAMQDKLGE
QIKTLEGEVETMNKRLHYLETTAKNSQEHIEKIIKGGGQS
Molecular Mass: 13290.77 g/mol

PFD2_His
Forward primer: GGAATTCCATATGCACCACCACCACCACCACATCGAAGGGCGTGCGACACAGTCTGCAGC
Reverse primer: CGGGATCCTCATGACTGCACGACCTGAACG
Expression vector: pET-23b
Expression host: *E coli.* BL21 star (DE3) pRARE
Complete amino-acid sequence of the construct produced:
MHHHHHHIEGRMATQSAAAKRQQDLQLQYTTYKNTLQQIAQKIGDVEQEAEEHKLVLETLEPLPGDRKCFRMINGVLVE
RTVQDVIPALRTNAEGLKKVLEDLVKQYKAKQDELEQWKKKNNVQVVQS
Molecular Mass: 14858.17 g/mol

PFD3_Strep
Forward primer: GGAATTCCATATGGCCAGTCAAGCAAAAGAAGC
Reverse primer: CTCGAGTTATTTTTCAAACTGTGGATGCGACCAACGCCCTTCGATTGAGCTGACGTCCCAGTTGTACAC
Expression vector: pET-23b
Expression host: *E coli.* Rosetta2 (DE3) pLysS
Complete amino-acid sequence of the construct produced:
MASQAKEAATKDATPSNPRGIPYAPFVDKVEDYVSSRDDVEPTLRRFQELIAKYQFMEQNLQRRVAGLKDKMPDIQKTLD
TVRFLKLRTSESDPIETTFELNDTLYAKAEIPPTDEVYLWLGANVMLAYPLDEAEQLLDSKLKAAKQSLANCEEDLDFLRE
QITTMEVAIARVYNWDVSSIEGRWSHPQFEK
21918.95 g/mol

PFD4
Forward primer: GGAATTCCATATGAACATAAGTCGGCGAATGGAG
Reverse primer: CGGGATCCTCATGTCTCGAGATTGATGGTCTTG
Expression vector: pET-23b
Expression host: *E coli.* Rosetta2 (DE3) pLysS
Complete amino-acid sequence of the construct produced:
MNISRRMEEEEAIGEDVEVRREDQDKINRFSRLHGHELAIEEELKAKQKEKEELDDITTELELADEDELVPYKVGDAFFHIP
LPQAQEMLGISTAKLEEEISQLEEKLSNIRDEMSQLKVELYARFGKTINLET
Molecular Mass: 15515.05 g/mol

PFD5
Forward primer: GGAATTCCATATGGCCAGCCGCAGC
Reverse primer: CGGGATCCTCATGAAGATTGCTTTGCTCCCG
Expression vector: pET-23b
Expression host: *E coli.* BL21 star (DE3) pRARE
Complete amino-acid sequence of the construct produced:
MASRSGQDKQGQGEAVDLASLSVQQLSTVKKQLDGEVEHLSTSYAQLVAAQGKFKECLRIVKDRASTLEKDKTVLVPLT
NSLYVKGKLSDPEHVLVDVGTGFYVEKDTTSAAEFYEGKIKELATNIQGLEGIVQAKTANLRVVEEVLRQKVLASNAAGT
GAKQSS
Molecular Mass: 17591.16 g/mol

PFD6
Forward primer: GGAATTCCATATGGCTGAAATCCAGGCCC
Reverse primer: CGGGATCCTCATGCCTTGACGGCTTGAAC
Cloning vector: pET-23b
Expression vector: pET-23b
Expression host: *E coli.* BL21 star (DE3) pRARE
Complete amino-acid sequence of the construct produced:
MAEIQARLQALSEEFSKLQQDLQDALQSRQKLEAQKQENLGVQKEFKKLKEGENIYKLIGPVLLKQDKVDAESIVNGRLD
FIDKEINRIETLIKELQGKIEKKKGEIIQVQSGLQAAAVQAVKA
Molecular Mass: 13879.64 g/mol
